# Characterization of Extended-Spectrum β-Lactamase-Producing *Escherichia coli* Isolates That Cause Diarrhea in Sheep in Northwest China

**DOI:** 10.1128/spectrum.01595-22

**Published:** 2022-08-09

**Authors:** Xueliang Zhao, Haoyu Zhao, Zilian Zhou, Yongqiang Miao, Ruichao Li, Baowei Yang, Chenyang Cao, Sa Xiao, Xinglong Wang, Haijin Liu, Juan Wang, Zengqi Yang

**Affiliations:** a College of Veterinary Medicine, Northwest A&F University, Yangling, Shaanxi, China; b College of Veterinary Medicine, Yangzhou University, Yangzhou, Jiangsu, China; c College of Food Science and Engineering, Northwest A&F University, Yangling, Shaanxi, China; Instituto de Higiene

**Keywords:** sheep, ESBL-producing *E. coli*, resistomes, biocide resistance, metal resistance, insert sequence

## Abstract

Development of extended-spectrum-β-lactamase (ESBL)-producing Escherichia coli is one the greatest threats faced by mankind. Among animals, chickens, pigs, and cattle are reservoirs of these pathogens worldwide. Nevertheless, there is a knowledge gap on ESBL-producing E. coli from small ruminants (i.e., sheep and goats) in China. The aim of this study was to identify and characterize the resistance profiles, resistomes, and sequence features of 67 ESBL-producing E. coli isolates from sheep in northwest China. The findings showed that *bla*_CTX-M_ and *bla*_TEM_ were the most prevalent. Interestingly, we found that the resistance gene *mcr-1* was widespread in sheep merely from Shaanxi areas, accounting for 19.2% (5/26). The highly prevalent serotypes and FumC-FimH (CH) typing isolates were O8 and C_4_H_32_, respectively. High-risk E. coli clones, such as sequence type 10 (ST10), ST23, ST44, and ST58, were also found in China’s sheep population. A total of 67 ESBL-producing isolates were divided into five phylogenetic groups, namely, B1 (*n* = 47, 70.1%), B2 (*n* = 1, 1.5%), C (*n* = 14, 20.9%), E (*n* = 1, 1.5%), and F (*n* = 1, 1.5%), with the phylogenetic groups for 3 isolates (4.5%) remaining unknown. Moreover, ESBL-producing E. coli isolates were also characterized by the abundance and diversity of biocide/metal resistance genes and insert sequences. We found that in ESBL-producing E. coli isolates, there were two different types of isolates, those containing ESBL genes or not, which led to large discrepancies between resistance phenotypes and resistomes. In summary, our study provides a comprehensive overview of resistance profiles and genome sequence features in ESBL-producing E. coli and highlights the possible role of sheep as antibiotic resistance gene disseminators into humans.

**IMPORTANCE** Antimicrobial resistance (AMR), especially the simultaneous resistance to several antibiotics (multidrug resistance [MDR]), is one of the greatest threats to global public health in the 21st century. Among animals, chickens, pigs, and cattle are reservoirs of these pathogens worldwide. Nevertheless, there is a knowledge gap on ESBL-producing E. coli from small ruminants in China. This study is the largest and most comprehensive analysis of ESBL-producing E. coli isolates from sheep, including antibiotic resistance profiles, phylogenetic groups, serotypes, multilocus sequence types (MLST), insert sequences (IS), antibiotic resistance genes, disinfectant resistance genes, and heavy metal resistance genes. We recommend extending the surveillance of AMR of sheep-origin E. coli to prevent future public health risks.

## INTRODUCTION

Antimicrobial resistance (AMR), especially the simultaneous resistance to several antibiotics (multidrug resistance [MDR]), is one of the greatest threats to global public health in the 21st century (1). Globally, by 2050, 10 million people a year will die from antibiotic-resistant bacteria according to estimates by the British government (2). By that time, MDR will be one of the major causes of death worldwide. The rapid growth in the MDR of Escherichia coli has been reported not only in ecological environment and human medicine but also widely in animal husbandry, particularly with an increasing prevalence of extended-spectrum beta-lactamases (ESBLs), which greatly compromises treatment effectiveness and increases morbidity and mortality (3, 4). ESBLs are plasmid-mediated enzymes that hydrolyze the broad-spectrum β-lactam ring, rendering the antimicrobial ineffective (5). Beta-lactamases are divided into four classes (A to D) based on their amino acid sequences (Ambler system). Class A (CTX-M, TEM, and SHV enzymes), C (CMY, DHA, and ACT enzymes), and D (OXA enzymes) beta-lactamases all rely on a key water molecule to hydrolyze β-lactam antibiotics, while class B beta-lactamases (metallo-β-lactamase enzymes) utilize zinc ions to attack the reactive β-lactam ring (6). Currently, the most common genetic type of ESBL is CTX-M (7). The CTX-M enzymes can be divided into five major groups: CTX-M-1, CTX-M-2, CTX-M-8, CTX-M-9, and CTX-M-25. The CTX-M family of ESBLs has emerged as the dominant mechanism of third-generation cephalosporins (e.g., ceftazidime, cefotaxime, ceftriaxone, and aztreonam) in E. coli (8).

ESBLs are considered an important cause of transferable MDR in E. coli because they are encoded mainly by plasmid and mobile genetic elements (MGEs). An increasing amount of data suggests that MGEs, including transposons, integrons, and insertion sequences, and plasmid replicon genotypes have played an important role in mobilizing *bla*_CTX-M_ genes, which assist ESBLs from animal transmission to other hosts (9, 10). Food-producing animals are an important reservoir of AMR (11). Over time, MDR to antibiotics has developed due to the selective pressure of the third-generation cephalosporins on E. coli. In recent years, the rapid increase in animal infections, especially with pathogenic bacteria, due to extended-spectrum cephalosporin resistance is particularly worrisome (12). The prevalences of ESBL-producing E. coli have been reported to be 78.6% in chicken, 70.7% in cattle, and 75.4% in swine (13, 14). Moreover, birds and dolphins might also play a key role in the development and intercontinental dissemination of ESBLs (15, 16). Among resistant E. coli isolates of animal origin, those from sheep have long been neglected, although a few previous studies have reported resistant E. coli from sheep (17). However, phenotypic and genotypic characterization of the E. coli isolates and the antibiotic resistance genes (ARGs) they carry in diarrheic sheep remain unknown.

As the cost of sequencing decreases, whole-genome sequencing (WGS) has become increasingly popular in characterizing bacterial isolates. WGS is commonly applied to analyze and understand the genotype and phenotype of drug-resistant bacteria (18). First, serotype, resistance genes, and phylogenetic and multilocus sequence typing (MLST) can be accurately predicted using WGS, thereby providing a better, faster, and cheaper analytical method (19). Furthermore, next-generation sequencing (NGS) data are the most accurate for identifying strains of a species, especially of those species with a high degree of genome homogeneity. Importantly, WGS-based online analysis tools have been developed and applied to the characterization of ESBL-producing E. coli isolates (20). Thus, to get a better insight into the characteristics of MDR ESBL-producing E. coli isolates from diarrheic sheep, the present study aimed at evaluating the resistant phenotypes, followed by WGS-based characterization, of E. coli isolates. To the best of our knowledge, this is the first report of ESBL-producing E. coli from sheep in China.

## RESULTS

### Antibiotic resistance profiles of E. coli.

**FIG 1:**
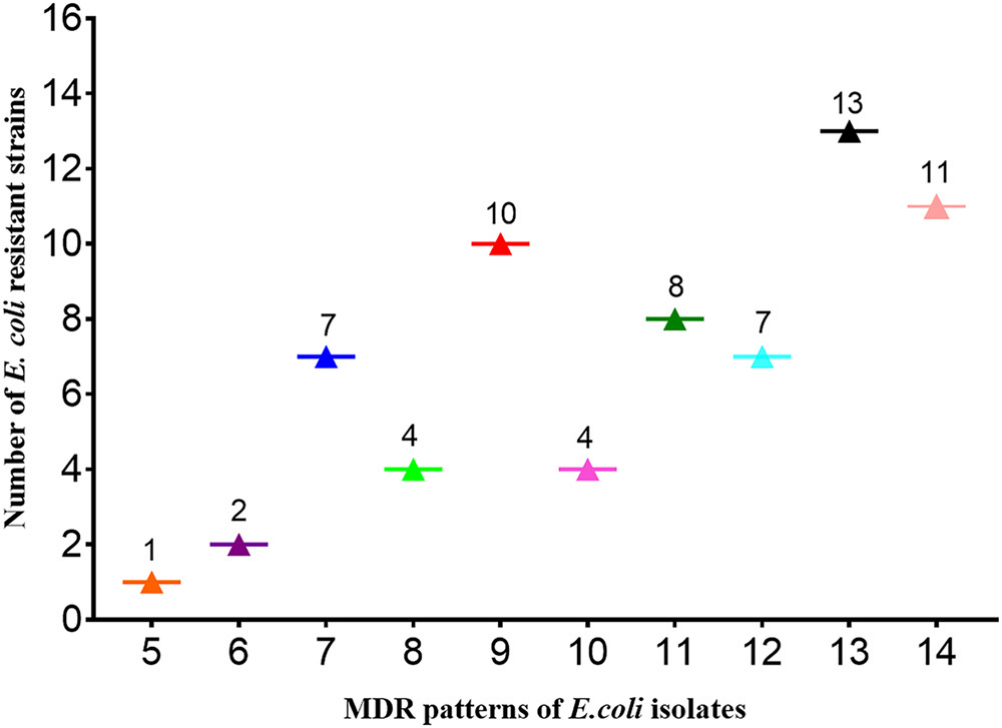
Antimicrobial resistance pattern and frequency of 67 MDR E. coli isolates.

Based on preliminary screening results from a previous study, we further confirmed 67 ESBL-producing isolates. Of these, 30 isolates were from Ningxia, 26 from Shaanxi, 8 from Qinghai, and 3 from Inner Mongolia. Table 1 indicates that 100% (67/67) of the isolates were resistant to ceftazidime, ceftiofur, ceftriaxone, and cefixime (third generation), while more than 38.8% (26/67) of the isolates were resistant to cefepime (fourth generation). In addition, the highest prevalence was for isolates with sulfisoxazole and florfenicol resistance, with a rate of 95.5% (64/67), followed by resistance to tetracyclines, mequindox, enrofloxacin, ampicillin, spectinomycin, gentamicin, and colistin, with rates of 80.6% (54/67), 76.1% (51/67), 76.1% (51/67), 70.1% (47/67), 68.7% (46/67), 55.2% (37/67), and 29.9% (20/67), respectively (Table 1). Of note, although all isolates were sensitive to meropenem, the trends of resistance cannot be ignored. A total of 67 E. coli isolates exhibited 10 MDR patterns, as they were resistant to at least five of the tested antibiotics. Most noticeably, 11 (16.4%) E. coli isolates in Shaanxi displayed resistance against 14 antibiotics (Fig. 1).

**TABLE 1:** MIC distribution of E. coli isolates and prevalence of MDR by isolate

																E. coli isolates (*n* = 67)					
	No. of isolates at indicated antimicrobial dilution (μg/mL)^*a*^															Sensitive		Intermediate		Resistant	
Antimicrobial	≤0.125	0.25	0.5	1	2	4	8	16	32	64	128	256	512	1,024	≥2,048	No.	%	No.	%	No.	%
Sulfisoxazole							0	0	0	0	0	0	3	21	43	0	0.0	3	4.5	64	95.5
Spectinomycin							0	2	6	13	9	13	5	11	8	8	11.9	13	19.4	46	68.7
Mequindox					0	1	3	12	6	12	9	7	17			4	6.0	12	17.9	51	76.1
Ampicillin					1	4	7	8	4	1	3	38	1			12	17.9	8	11.9	47	70.1
Gentamycin				4	12	11	3	2	2	4	4	25				27	40.3	3	4.5	37	55.2
Tetracyclines				2	5	3	3	7	10	18	7	12				10	14.9	3	4.5	54	80.6
Florfenicol				0	0	1	7	5	11	4	5	34				1	1.5	7	10.4	59	88.1
Ceftazidime				0	0	0	0	21	18	13	9	6				0	0.0	0	0.0	67	100
Cefepime			25	4	9	2	1	2	1	2	21					38	56.7	3	4.5	26	38.8
Ceftiofur			0	0	0	0	5	6	14	6	36					0	0.0	0	0.0	67	100
Ceftriaxone		0	0	0	0	38	25	17	12	7						0	0.0	0	0.0	67	100
Cefixime		0	0	0	0	25	16	9	6	11						0	0.0	0	0.0	67	100
Meropenem	50	3	5	5	4	0	0	0	0							63	94.0	4	6.0	0	0.0
Colistin	17	15	8	6	1	2	0	0	18							46	68.6	1	1.5	20	29.9
Enrofloxacin	4	1	2	9	6	3	1	3	38							7	10.4	9	13.4	51	76.1

a Shaded values indicate the numbers of isolates at antimicrobial dilution breakpoints; all other values are the numbers of isolates at applied antimicrobial dilutions.

### Phylogenetic groups and serotypes for the ESBL-producing E. coli isolates.

A total of 67 ESBL-producing isolates were divided into five phylogenetic groups, namely, B1 (*n* = 47, 70.1%), B2 (*n* = 1, 1.5%), C (*n* = 14, 20.9%), E (*n* = 1, 1.5%), and F (*n* = 1, 1.5%), with the phylogenetic groups for 3 isolates (4.5%) remaining unknown. There were no representatives from phylogenetic group A, D, or F or from clade I.

Next, we further evaluated ESBL-producing E. coli isolate serotypes. A total of 33 serotypes were identified using SerotypeFinder 2.0, whereas in 5 genomes, only the H antigen was identified. The most frequent serotypes were O8 (*n* = 8), O9 (*n* = 6), O18 (*n* = 4), O89 (*n* = 4), O101 (*n* = 4), O185 (*n* = 4), O21 (*n* = 3), and O45 (*n* = 3), which have been previously reported to cause diarrhea. In our study, multiple serotypes were observed in the E. coli isolates, and for prevalence estimates, only one serotype was counted. All of the serotype O9 (*n* = 6), O18 (*n* = 4), and O89 (*n* = 4) isolates harbored ESBL genes. ESBL-producing E. coli isolates were serotyped to determine their association with known pathogenic serotypes. Fortunately, the O157 serotype was not detected in sheep-origin E. coli. Strikingly, using PathogenFinder, we predicted that the probability of 67 E. coli isolates as a human pathogen was 92.2% to 94.6%. A total of 486 to 774 pathogenic families were matched to 67 E. coli isolate genomes. That is, all isolates of ESBL-producing E. coli isolates were pathogenic.

### Multilocus sequence typing (MLST) and FumC-FimH (CH) typing.

**FIG 2:**
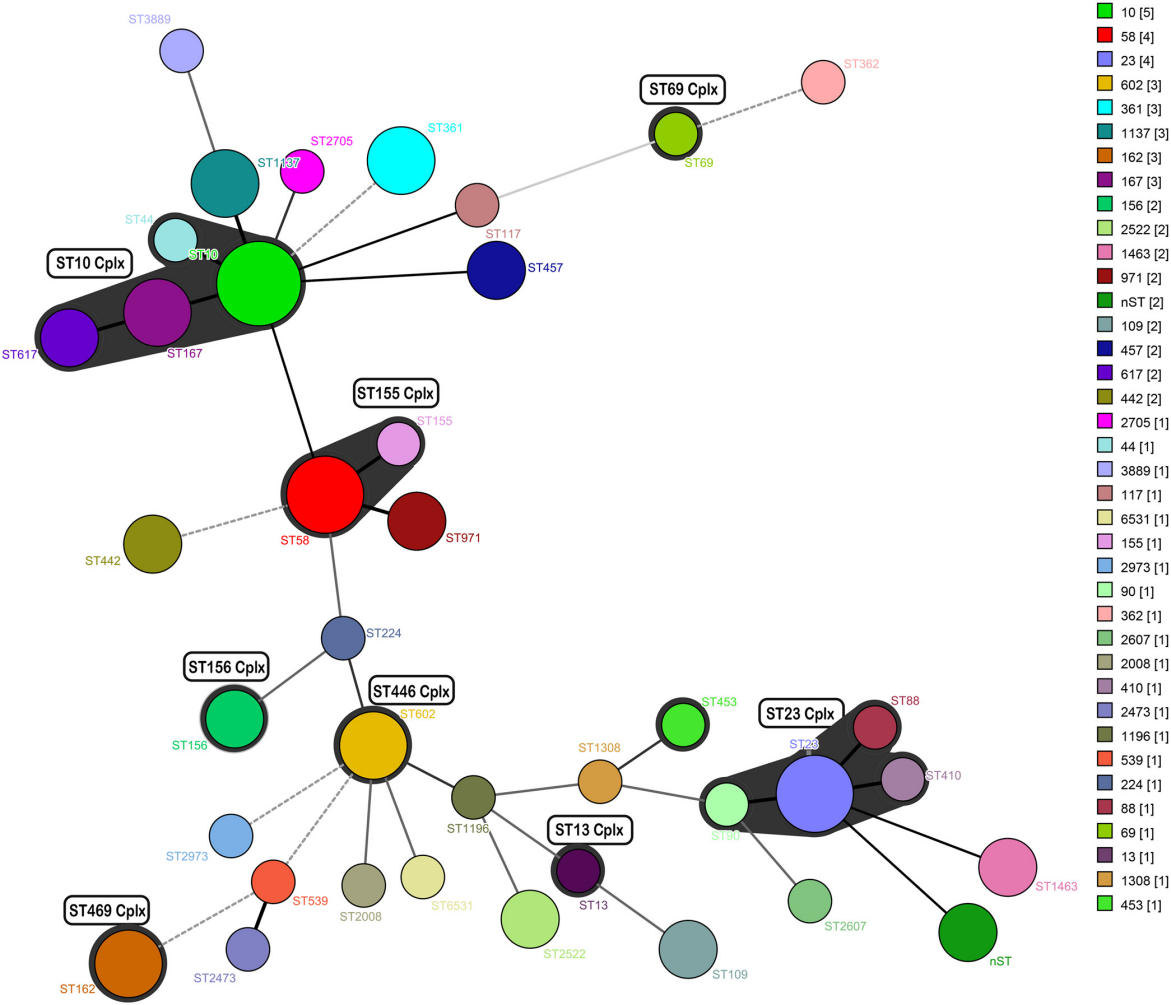
Identification of the molecular epidemiology 67 ESBL-producing E. coli isolates of sheep origin. The minimum spanning tree was obtained using BioNumerics v7.6 software. Each circle represents a special sequence type (ST), and the size of the circle indicates the number of isolates in that ST. The distance labels correspond to the number of discriminating alleles.

In this study, ESBL-producing isolates were assigned to 37 different STs. Of these, 8 STs (i.e., ST10, ST23, ST58, ST162, ST167, ST361, ST602, and ST1137), with more than three strains in each ST, were recognized as the dominant STs. For three STs (i.e., ST162, ST361, and ST602), all isolates harbored ESBLs-encoding genes. For the STs that contained more than two isolates, the majority of E. coli strains had the same serotype. A total of 65 strains (97.0%) had combinations of known ST alleles, while 2 strains (3.0%) had novel ST alleles resulting in previously unknown ST types. We further assessed the clonality and clades of all 67 ESBL-producing isolates as minimum spanning trees by BioNumerics 7.6 (Fig. 2). Our results showed the substantial horizontal dissemination of ESBLs through sheep-origin E. coli.

Furthermore, according to CH typing, unique combinations of *fumC* and *fimH* alleles are used to identify the E. coli isolates. By using genome analysis undertaken with CHTyper, we identified 22 *fimH* alleles and 13 *fumC* alleles. Ten types were detected by CH typing, with the most frequent being FumC4/FimH32 in 10 of 67 isolates (14.9%). Among the 67 total isolates, the most frequent *fimH* alleles were H32 (23.9%, *n* = 16), H31 (7.5%, *n* = 5), H38 (7.5%, *n* = 5), and H86 (7.5%, *n* = 5), while the most frequent *fumC* alleles were C4 (26.9%, *n* = 18), C11 (22.4%, *n* = 15), and C6 (10.4%, *n* = 7). The phylogenetic tree based on single nucleotide polymorphisms (SNPs) in the core genome showed distinct clustering of strains carrying specific *fimH*-*fumC* alleles. Interestingly, some CH typings were closely related to specific serotypes and STs. Strains with the same serotype and ST typically harbor the same CH typing.

### Characterization of the ESBL genes in E. coli isolates.

Forty-three isolates (64.2%) showed coexistences of more than one gene, with 49.3% (33/67) of ESBL-producing isolates harboring *bla*_CTX-M_, *bla*_TEM_, and *bla*_OXA_. *bla*_SHV_, *bla*_CMY_, and *bla*_KPC_ genes were not detected in isolates, and 50.7% (34/67) of the ESBL-producing isolates were negative for ESBL-encoding genes. However, all of the ESBL-producing isolates harbored *bla*Ec. *bla*_CTX-M_ genes were detected in 26 of 67 ESBL-producing isolates: 16 (61.5%) were *bla*_CTX-M-55_, 5 (19.2%) were *bla*_CTX-M-15_, 3 (11.5%) were *bla*_CTX-M-14_, 2 were (7.7%) *bla*_CTX-M-65_, 1 (3.8%) was *bla*_CTX-M-17_, and one O224:H23 isolate (SX15) harbored both the *bla*_CTX-M-55_ and *bla*_CTX-M-65_ genes. Among the 26 isolates harboring *bla*_CTX-M_, only 1 isolate contained *bla*_CTX-M_ alone, while the remaining 25 isolates harbored *bla*_CTX-M_ in combination with *bla*_TEM_ and/or *bla*_OXA_ genes. A portion of ESBL-producing isolates (38.8%) harbored *bla*_TEM_ genes, of which 20 harbored *bla*_TEM-1_, 5 harbored *bla*_TEM-150_, and one harbored *bla*_TEM-235_. Of the 67 isolates, 6 isolates were positive for *bla*_OXA-1_ and 1 isolate was positive for *bla*_OXA-10_. Interestingly, 78.9% (26/33) of the isolates contained two resistance genes (*bla*_CTX-M_ and *bla*_TEM_, *bla*_TEM_ and *bla*_SHV_, or *bla*_CTX-M_ and *bla*_OXA_), and 6.0% (2/33) of the isolates concurrently contained *bla*_CTX-M_, *bla*_TEM_, and *bla*_OXA_.

### ESBL-producing E. coli isolates carried a wide range of resistomes and disinfectant and heavy metal resistance genes.

We also analyzed 67 sequenced samples for multiple antimicrobial resistance genes (ARGs). Of all the ARGs analyzed, 29 ARG types were detected in this study, and of these 29 ARG types, 10 (34.5% of the detected ARGs) were found in at least 20 of the 67 isolates and could be considered “widespread.” These included aminoglycoside resistance genes [*aph(3′)-Ia* (*n* = 12), *aph(3″)-Ib* (*n* = 29), *aph(4)-Ia* (*n* = 3), *aph(6)-Id* (*n* = 30), *aac(3)-IId* (*n* = 13), *aac(3)-IIe* (*n* = 8), *aac(3)-IVa* (*n* = 4), *aac(6′)-Ib* (*n* = 6), and *rmtB1* (*n* = 1)], aminoglycoside resistance cassettes [*aadA1* (*n* = 4), *aadA2* (*n* = 12), *aadA5* (*n* = 11), *aadA16* (*n* = 1), and *aadA22* (*n* = 2)], tetracycline resistance genes [*tet*(A) (*n* = 28), *tet*(B) (*n* = 16), *tet*(M) (*n* = 3), and *tet*(34) (*n* = 14)], sulfonamide resistance genes [*sul1* (*n* = 18), *sul2* (*n* = 29), and *sul3* (*n* = 5)], trimethoprim resistance genes [*dfrA1* (*n* = 2), *dfrA12* (*n* = 10), *dfrA14* (*n* = 5), *dfrA17* (*n* = 12), and *dfrA27* (*n* = 1)], quinolone resistance genes [*oqxA* (*n* = 3), *oqxA2* (*n* = 2), *oqxB* (*n* = 7), and *qepA4* (*n* = 1)], chloramphenicol resistance genes [*cmlA1* (*n* = 3), *cmlA5* (*n* = 1), *cmlA6* (*n* = 1), *catA1* (*n* = 5), *catA2* (*n* = 1), and *catA3* (*n* = 3)], a florfenicol resistance gene [*floR* (*n* = 19)], and an azithromycin resistance gene [*mph*(A) (*n* = 19)]. Furthermore, a set of resistance genes to macrolide [*erm*(B) (*n* = 5) and *erm*(42) (*n* = 2)], fosfomycin [*fosA* (*n* = 7)], bleomycin [*bleO* (*n* = 5)], rifampin [*arr-2* (*n* = 2) and *arr-3* (*n* = 2)], and lincosamide [*lnu*(F) (*n* = 2)] was rarely observed in our study. Interestingly, 84.6% of the ARGs were found in Shaanxi, while only 26.8% were detected in other regions. Surprisingly, 5 isolates harboring the *mcr-1* gene were detected in Shaanxi. To the best of our knowledge, this is the first report of ESBL-producing E. coli harboring the colistin resistance gene *mcr-1* in wool sheep in China.

**FIG 3:**
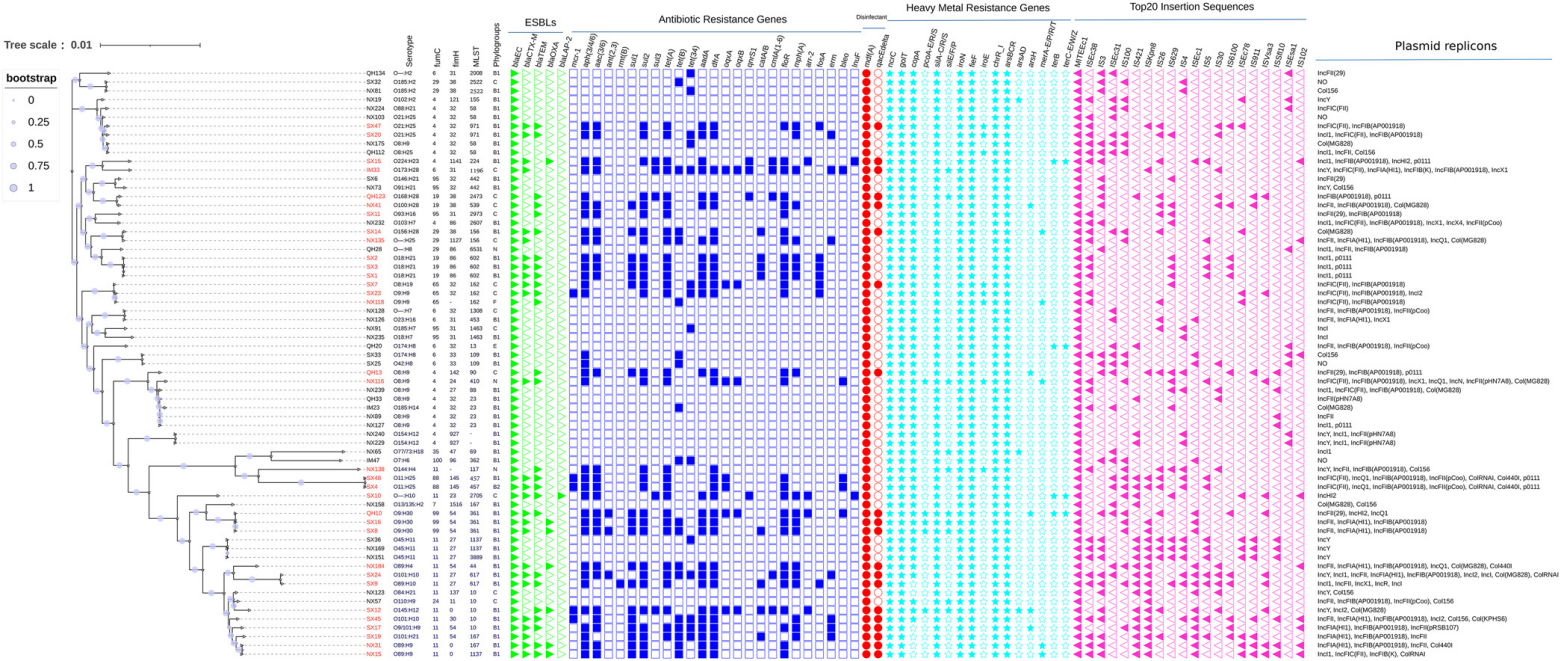
Evolutionary tree analysis of the 67 ESBL-producing Escherichia coli strains sequenced in this study. Core genome alignments were obtained using the Roary program, and a maximum likelihood tree was assembled using the FastTree program. Visualization and annotation were carried out through iTOL version 6.52 (https://itol.embl.de; accessed 6 March 2022). Phylogenetic groups, MLST, O and H serotypes, *fumC*-*fimH* typing, IS, and the resistance genes of antibiotics, disinfectants, and heavy metals were plotted. Phylogenetic analysis showed a relatively well-supported monophyletic distribution of STs. A dash in the serotypes, *fimH*, and sequence type (ST) columns indicates an unrecognized serotype, *fimH* type, and ST in the corresponding database.

We predicted a large collection of genes associated with biocide and metal resistance genes against the BacMet database. In terms of heavy metal resistance genes, the *qacEΔ1* gene was detected in 20 of 67 (29.8 %) isolates. Surprisingly, in terms of heavy metal resistance genes, ESBL-producing E. coli strains harbored the intact nickel resistance gene *ncrC* (*n* = 67, 100%), the gold resistance gene *golT* (*n* = 67, 100%), silver resistance genes *sliABCRS* (*n* = 67, 100%), the iron resistance gene *fieF* (*n* = 67, 100%), the chromium resistance gene *chrR* (*n* = 67, 100%), and arsenic resistance genes *arsCBR* (*n* = 67, 100%). Furthermore, genes associated with resistance to copper (*copA*, *pcoA*, *pcoB*, *pcoC*, *pcoD*, *pcoR*, *pcoS*, and *pcoE*), silver (*silE*, *silF*, and *silP*), iron (*iroE*), arsenic (*arsA*, *arsD*, and *arsH*), mercury (*merA*, *merC*, *merD*, *merE*, *merT*, *merR_Ps*, and *merP_Gneg*), and tellurium (*terB*, *terC*, *terD*, *terE*, *terW*, and *terZ*) were also detected (Fig. 3).

### Mobile genetic elements associated with antimicrobial resistance.

Mobile genetic elements play an important role in facilitating horizontal genetic exchange and therefore promote the acquisition and spread of resistance genes. Insertion sequences (IS) mediate resistance to antibiotics, heavy metals, and disinfectants. We analyzed mobile genetic elements and insertion sequences using MobileElement Finder. Seventy-five IS were identified in this study. Specifically, each strain contained the IS ITEEc1 (*n* = 67). The top 10 IS were MITEEc1, ISEc38, IS*3*, ISEc31, IS*100*, IS*421*, ISKpn8, IS*26*, IS*629*, IS*4*, and ISEc1 (Fig. 3). Of the 67 isolates, 19 harbored more than 10 IS, and to our surprise, 13 of these 19 were from Shaanxi Province (see File S1 in the supplemental material). As the presence of numerous IS might be one of the major causes of MDR to E. coli, plasmid replication types were also identified using PlasmidFinder 2.1. In total, 26 different plasmid replicon types were predicted. The predominantly found plasmid replicon types were of the IncFIB (AP001918) and IncFII, at 47.8% and 28.4%, respectively. In four ESBL-producing E. coli isolates, however, no plasmid replicons could be predicted by the PlasmidFinder. Various replicon types carried by these plasmids highly challenge the antimicrobial resistance.

### MDR rate between harbored ESBL genes and non-ESBL genes.

**FIG 4:**
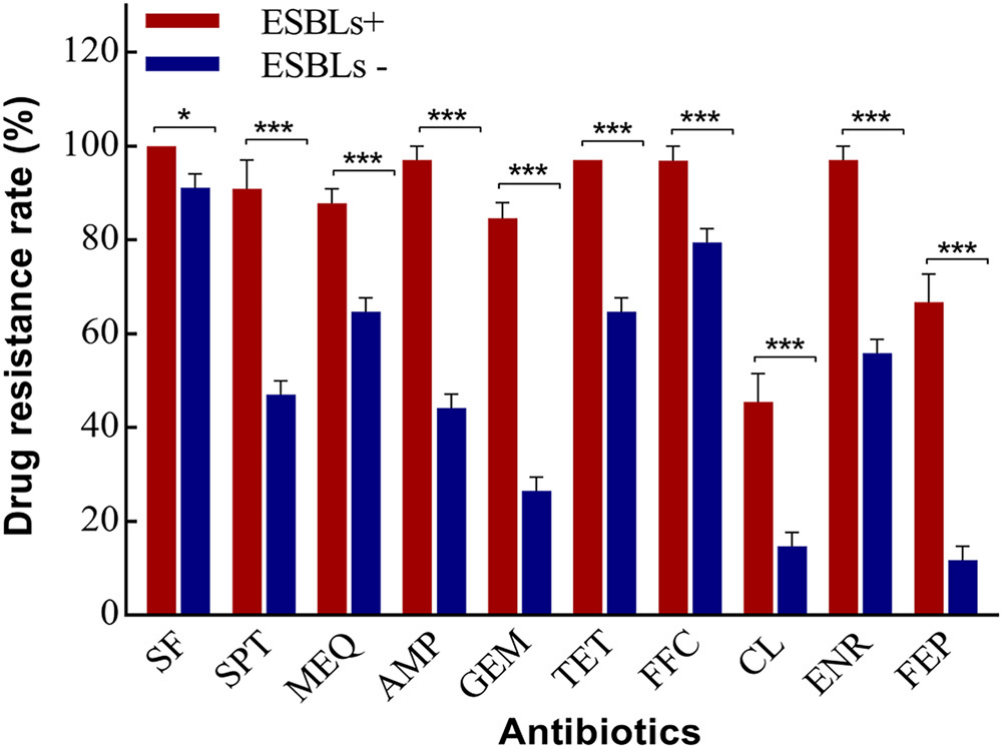
MDR rate between isolates containing targeted ESBL genes (ESBLs+) and not containing ESBL genes (ESBLs−).

It should be noted that despite the fact that all 67 isolates exhibited the ESBL-producing phenotype, only 33 isolates were genotypically confirmed to harbor ESBL resistance genes (*bla*_CTX-M_, *bla*_TEM_, or *bla*_OXA_). The ESBL-producing E. coli isolates were divided into the ESBL group and the non-ESBL group according to whether they harbored ESBL genes. Of the 67 E. coli isolates, the ESBL group comprised 33 (49.3%) strains and the non-ESBL group consisted of 34 (50.7%) strains. The detection rates in strains of MDR to sulfisoxazole, spectinomycin, mequindox, ampicillin, gentamicin, tetracyclines, florfenicol, colistin, enrofloxacin, and cefepime in 33 ESBL-producing E. coli isolates were 100.0%, 90.9%, 87.9%, 97.0%, 84.8%, 97.0%, 97.0%, 45.5%, 97.0%, and 66.7%, respectively. In addition, MDR of ESBL group isolates was significantly higher than that of the non-ESBL group for 10 antibiotic drug classes (Fig. 4).

## DISCUSSION

Animals, particularly food animals, have long been recognized as important reservoirs for ESBL-producing E. coli isolates (21). However, little research has been reported about multiple-antibiotic- resistant E. coli in sheep. In recent years, with the extensive application of antibiotics (such as β-lactams), including abuse and misuse of antibiotics (22), the high levels of the drug resistance phenomenon in sheep-origin E. coli have increased (23). One of the aims of this study was to reveal a wide range of multiple-antibiotic resistance profiles of ESBL-producing E. coli isolates. The present study reports the antibiotic resistance profiles of 67 ESBL-producing E. coli strains isolated from diarrheic sheep. The emergence of E. coli isolates conferring resistance to third/fourth-generation cephalosporin and colistin from wool sheep in this study is alarming, because third/fourth-generation cephalosporin and colistin are not licensed for use in sheep worldwide. Further, these isolates were also resistant to sulfisoxazole, tetracyclines, and florfenicol, and none of them were resistant to meropenem. In addition, 33 strains containing ESBL genes showed a worryingly high level of resistance to third/fourth-generation drugs, which should attract much attention. High levels of multidrug resistance were observed, as 100% of strains were resistant to at least five antibiotics, 64.2% of strains showed resistance to more than 10 antibiotics, and 16.47% showed resistance to as many as 14 antibiotics. This is a startling discovery, as until now, sheep-origin E. coli resistance has been neglected. Recent studies have confirmed the presence of MDR ESBL-producing E. coli in sheep in Pakistan, Chile, Brazil, Portugal, and the United States (24–28). To the best of our knowledge, this is the first report of the multidrug resistance determinants of ESBL-producing E. coli isolates of sheep origin in China.

Phylogenetic analysis showed that the 67 ESBL-producing E. coli isolates belonged to five phylogroups: B1, B2, C, E, and F. Phylogenetic groups B1 and C comprised more than half (90%) of the total E. coli isolates from diarrheic sheep in our study. However, in a previous study, a dominance of group A or B1 was observed in ESBL-producing E. coli of sheep origin, in contrast to the results in our study. For example, a study in Portugal showed that phylogroups B1 and A comprised 92.6% of the total E. coli isolated from sheep, although the proportion of B1 was about twice the proportion of phylogroup A in their study (29), similar to our results. Thus, the distribution of phylogenetic groups may be influenced by host species, geographical regions, climate, and so on. Notably, it was previously reported that phylogenetic group B1 isolates were said to be associated with extraintestinal pathogenic E. coli (ExPEC) infections in humans and animals (30, 31). To further confirm these results, we predicted the probability that the 67 E. coli isolates were human pathogens by PathogenFinder. Unexpectedly, all the isolates of ESBL-producing E. coli were pathogenic. Among the 33 different serotypes of ESBL-producing E. coli detected in our study, one was O8, the most common serogroup of enterotoxigenic E. coli (ETEC) capable of causing disease in other animals. In addition, serotypes O8, O9, O18, O89, O101, and O185 accounted for a high proportion and O18 was the predominant serotype among the ESBL-producing E. coli isolates. Most of the serotypes (28/33) were found pathogenic for animals and even humans in previous studies. Thus, it is reasonable to assume that most of the ESBL-producing E. coli strains found in sheep were pathogenic for humans in this study. However, investigations into the pathogenic potential of ESBL-producing E. coli are required in our future studies.

MLST is an accurate and expansive molecular typing method which has been used for typing and establishing clonal relationships between E. coli isolates. Our results showed that 67 ESBL-producing E. coli isolates belonged to 37 STs, with ST10 (*n* = 5), ST23 (*n* = 4), and ST58 (*n* = 4) being the most prevalent types. Among these, at least nine of the STs (ST10, ST23, ST44, ST58, ST69, ST455, ST90, ST162, and ST361) were previously reported in ESBL-producing E. coli (32–35). For instance, ESBL-producing E. coli of ST361, ST167, and ST69 have been reported in Denmark, while ST10 and ST69 have been reported in Switzerland (36). Interestingly, ST167 and ST1137 isolates harbored three unique types of ESBL genes (*bla*_CTX-M-15_, *bla*_TEM-150_, and *bla*_OXA-1_). Furthermore, to our surprise, ST167, ST10, and ST23 were generally considered to be associated with infections related to humans (37, 38). Our findings showed that ST167, ST10, and ST23 strains were highly detected in sheep, which may account for the success of ST167, ST10, and ST23 as emerging pathogens. On the other hand, we combined conventional *fumC*-*fimH* typing with second-generation sequencing to assess E. coli clonal diversity. Notably, the conclusion of our study indicated that the epidemic FumC4/FimH32 type might dominate in ESBL-producing E. coli. However, ST131-H30, a pandemic, multidrug-resistant, and highly pathogenic E. coli subclone, was not detected in our study.

Over the past 25 years, CTX-M β-lactamases have become the most widely distributed ESBLs in E. coli infections globally. *bla*_CTX-M-15_ has long been considered the most frequent of CTX-M type β-lactamases (39). However, the most predominant CTX-M type genes detected in this study were *bla*_CTX-M-55_ and *bla*_CTX-M-15_, followed by *bla*_CTX-M-14_, *bla*_CTX-M-65_, and *bla*_CTX-M-17_. *bla*_CTX-M-15_ was reported as the most frequent ESBL gene in E. coli from patients (40), animals (41), and environments (42). Recently, studies revealed that *bla*_CTX-M-55_ has increased significantly in animals, retail raw meat, and patients, suggesting a rapid dissemination of *bla*_CTX-M-55_ (43–45) In this study, we obtained similar conclusions: the detection rate of *bla*_CTX-M-55_-positive isolates reached 61.5% (16/26), which was higher than that reported in previous research studies. Most *bla*_CTX-M_-positive isolates have been reported as coharboring *bla*_TEM_, and similar results were obtained in our study. As with *bla*_CTX-M-55_, *bla*_TEM-1_ plays a significant role in ESBL-producing E. coli of diarrheic sheep. Moreover, we also found two isolates that harbored *bla*_CTX-M_, *bla*_TEM_, and *bla*_OXA_ genes at the same time. Overall, 84.9% (28/33) of the strains harbored two or three ESBL-producing genes, which indicates isolates containing multiple ESBL resistance genes. Moreover, ESBL-producing E. coli isolates from sheep carried AMR conferring resistance to sulfisoxazole, spectinomycin, mequindox, ampicillin, gentamicin, tetracyclines, florfenicol, colistin, and enrofloxacin. It is worth mentioning that five isolates from Shaanxi harbored the *mcr-1* gene. This is the first report of ESBL-producing E. coli carrying the *mcr-1* gene in sheep, as well as possessing the rare sulfonamide resistance gene *sul3*. We were also the first to identify the 16S rRNA methylase gene *rmtB1* from ESBL-producing E. coli that showed very high levels of resistance to aminoglycoside. Furthermore, we found plasmid-mediated fosfomycin resistance glutathione transferase genes *fosA3* and *fosA7.5*, type A1/2 chloramphenicol *O*-acetyltransferase gene *catA1/2*, chloramphenicol efflux MFS transporter gene *cmlA1/5/6*, and fluoroquinolone efflux MFS transporter gene *qepA4*. The presence of ESBL-producing E. coli isolates carrying colistin resistance gene *mcr-1*, aminoglycoside resistance gene *rmtB1*, and sulfisoxazole resistance genes *sul1/2/3* from sheep aroused great public health concern, as therapeutic choices in such cases are very limited. Our study detected AMR in ESBL-producing E. coli of sheep that was more diversified than previously reported for sheep in Portugal (27). Notably, all isolates in our study were resistant to multiple antibiotics, meaning that existing treatment protocols are unlikely to be curative.

Biocide and metal resistance genes were common in ESBL-producing E. coli isolates of sheep. In this study, ESBL-producing E. coli harbored a broad resistance gene against disinfectants (*qacEΔ* and *mdfA*) and heavy metals (nickel, gold, copper, silver, iron, chromium, arsenic, and mercury). This is line with previous disinfectant studies, which showed that the widespread use of disinfectants may cause strong selection pressure, giving rise to the appearance of cross-resistance and coresistance between strains for disinfectants and antimicrobial agents. Previous studies have shown that disinfectants can also lead to the development of disinfectant-resistant E. coli and might not eliminate resistant E. coli (46). As previously mentioned, the use of antibiotics may inadvertently promote enhanced resistance to antimicrobial metals (47). Likewise, ESBL-producing E. coli isolates contained a large cluster of metal resistance genes in our study. Therefore, careful consideration is necessary when selecting disinfectants and antibiotics.

In this study, despite the fact that all ESBL-producing E. coli isolates harbored a *bla*Ec gene, targeted ESBL genes were not detected in 34 of 67 (50.7%) isolates. In contrast, the rates of resistance to the other 10 antibiotics in the ESBL gene groups were significantly higher than those in the non-ESBL gene groups. Interestingly, the prevalence of antibiotic resistance genes was higher in the ESBL gene group than in the non-ESBL gene group, and the rapid rise of ESBLs apparently also increased the selective pressure of antibiotic resistance. Of the ESBL gene-positive E. coli isolates, all had at least 4 resistance genes, and several isolates carried 20 or more resistance genes. We presumed that the ESBL gene-positive isolates were more likely to recruit the resistance genes than isolates without target ESBL genes. Considering that *bla*_CTX-M-55_ has become increasingly prevalent in ESBL-producing E. coli isolates of animal origin but is relatively rare in isolates of human origin, this finding might indicate that *bla*_CTX-M-55_ and a variety of drug resistance genes emerged and rose under antibiotic selective pressure in animal husbandry (21). Therefore, prohibiting or strictly curtailing antimicrobial use in animal husbandry is urgently needed to address the increasing threat of antimicrobial resistance. Furthermore, results from this study indicate that the ESBL-producing E. coli resistance mechanism might be complex. In the absence of specific drug resistance genes, multiple drug metabolism pathways may exist on the basis of multiple drug resistance phenotypes.

Despite this being the largest and most comprehensive analysis of ESBL-producing E. coli from sheep, several limitations still exist in this study. First, for the sampling, we selected only two cities per province, which may not represent provincial conditions entirely. Second, the sample size from free-range sheep was not large enough, which might cause statistical errors. These limitations notwithstanding, our study provides new evidence on a China-wide scale for wool sheep as a potential reservoir for antibiotic resistance determinants. Moreover, the genomes of 67 E. coli strains were sequenced and analyzed through a bioinformatics approach. To the best of our knowledge, this is the most comprehensive characterization to date of antibiotic resistance profiles, phylogenetic groups, serotype, MLST, IS, antibiotic resistance genes, disinfectant resistance genes, and heavy metal resistance genes of ESBL-producing E. coli in wool sheep. We recommend extending surveillance of AMR of sheep-origin E. coli to prevent future public health risks.

## MATERIALS AND METHODS

### Bacterial isolation and antimicrobial susceptibility testing.

A total of 67 E. coli strains were isolated from a previous study (17). Briefly, these isolates were collected from cloacal swabbing of sheep in Shaanxi, Ningxia, Inner Mongolia, and Qinghai of China between 2019 and 2020. According to the guidelines of the Clinical and Laboratory Standards Institute (CLSI, 2020), ESBL-producing isolates were screened and confirmed for ESBLs production by the MICs and double disk synergy test, respectively. Fifteen antibiotics, namely, sulfisoxazole, spectinomycin, mequindox, ampicillin, gentamicin, tetracyclines, florfenicol, ceftazidime, cefepime, ceftiofur, ceftriaxone, cefixime, meropenem, colistin, and enrofloxacin were tested for MIC using the broth microdilution method. Each test was repeated three times per strain and using E. coli ATCC 25922 as a quality control strain. The confirmed ESBL-producing isolates were used for further analysis.

### Phylogenetic grouping.

Phylogenetic groups (A, B1, B2, C, D, E, or F) were identified according to the updated multiplex PCR in accordance with the scheme of Clermont et al. (48). Briefly, DNA from 67 E. coli isolates was subjected to quadruplex PCR targeting three genes (*chuA*, *yjaA*, and *arpA*) and a DNA fragment (TspE4.C2). All testing was performed with positive and negative controls.

### WGS.

For genotypic characterization, whole-genome sequencing (WGS) was performed on 67 ESBL-producing E. coli isolates. The total genomic DNA of these isolates was constructed using a purification kit (TianGen, Beijing, China) and then subjected to WGS. The next-generation sequencing library was constructed using a NEBNext Ultra DNA library prep kit (New England Biolabs, Ipswich, UK) according to the manufacturer’s protocol, and 150-bp paired-end reads were obtained from an Illumina HiSeq4000 platform. Illumina raw reads were assembled using SPAdes version 3.14.0. (http://bioinf.spbau.ru/spades) (49).

### Bioinformatics analysis.

Genome assembly qualities were assessed using QUAST (http://quast.sourceforge.net/quast). The assembled sequences were annotated using the rast (https://rast.nmpdr.org/rast.cgi) and prokka (https://github.com/tseemann/prokka) automatically (50, 51). Serotype, antimicrobial resistance genes, and FimH/FumC were identified using SerotypeFinder, ResFinder, and CHTyper tools, respectively, from Center for Genomic Epidemiology (http://www.genomicepidemiology.org/). Insertion sequences and plasmid replicon genotype were also identified using MobileElement Finder (https://cge.food.dtu.dk/services/MobileElementFinder/) and PlasmidFinder 2.1 (https://cge.food.dtu.dk/services/PlasmidFinder/), respectively (52, 53). BacMet (http://bacmet.biomedicine.gu.se/) was used to identify biocide and metal resistance genes associated with non-antibiotic elements (54). Multilocus sequence typing (https://cge.food.dtu.dk/services/MLST/) of ESBL-producing E. coli isolates was performed via MLST v2.0 database (55). Visualization of E. coli core genome MLST allelic profiles was built using BioNumerics 7.6. PathogenFinder (https://cge.food.dtu.dk/services/PathogenFinder/) was used to predict the pathogenic potential toward humans in the E. coli genomes (56). In addition, the evolutionary tree was constructed using maximum likelihood (Roary and FastTree) based on single nucleotide polymorphisms (SNPs) of the core genomes. In this study, the Interactive Tree of Life (https://itol.embl.de) Web server was used for the graphic visualization of the phylogenetic tree with the corresponding serotype, ST, FimH/FumC, ARGS, and insertion sequences in this study.

### Data availability.

All data can be requested from the corresponding author(s). All sequencing data are available at NCBI (BioProject accession no. PRJNA857498).
